# Antimicrobial resistance and molecular characterization of *Escherichia coli* isolated from bovine mastitis samples in Nghe An province, Vietnam

**DOI:** 10.14202/vetworld.2023.743-751

**Published:** 2023-04-13

**Authors:** Tran Trung My, Le Van Thien, Vu Duy Manh, Bui Thi Phuong My, Dang Thi Mai Lan, Dang Xuan Binh, Vu Minh Duc

**Affiliations:** 1 Department of Animal Science and Veterinary Medicine, Thai Nguyen University of Agriculture and Forestry, Thai Nguyen University, Quyet Thang, Thai Nguyen City, Vietnam; 2 Department of Quality Assurance, TH Dairy Institute, Nghia Son, Nghia Dan, Nghe An, Vietnam; 3 Department of Veterinary Medicine, The Vietnam National University of Agriculture, Trau Quy, Gia Lam, Ha Noi, Vietnam; 4 TH Milk Food Joint Stock Company, Nghia Son, Nghia Dan, Nghe An, Vietnam; 5 Department of Agro-forestry Technology, College of Economics and Technology, Thai Nguyen University, Thinh Dan, Thai Nguyen City, Vietnam; 6 Laboratory of Veterinary Public Health, Department of Veterinary Medicine, Joint Faculty of Veterinary Medicine, Kagoshima University, 1-21-24 Korimoto, Kagoshima 890-0065, Japan

**Keywords:** antimicrobial resistance, bovine mastitis, *Escherichia coli*, virulence genes

## Abstract

**Background and Aim:**

Vietnam’s dairy sector is in its early phase of large-scale farming development. Therefore, mastitis in cows is always a concern to farm owners. This study aimed to determine the antimicrobial susceptibility, resistance, and virulence-related genes of *Escherichia coli* isolated from bovine mastitis in Nghe An province of Vietnam.

**Materials and Methods:**

Fifty *E. coli* strains were isolated from the clinical cases and subjected to this study. All isolates were tested for antimicrobial susceptibility by the disk-diffusion method, as described by the Clinical and Laboratory Standards Institute. Antimicrobial and virulence genes were confirmed by polymerase chain reaction with specific primers.

**Results:**

All isolates were resistant to lincomycin and sulfamethoxazole and sensitive to gentamicin, while other antimicrobials showed resistance from 2% to 90%. Multidrug resistance was confirmed in 46% of isolates, and none of them were identified as extended-spectrum beta-lactamase producers. From fifty strains tested for antimicrobial and virulence genes, six isolates harbored *tet*A, 6 *tet*B, 13 *sul*1, 15 *sul*2, 2 Intimin (*eae*), 1 *iut*A, and 3 *stx*2.

**Conclusion:**

Antimicrobial and multidrug resistances are the main virulence factors of *E. coli* isolated from bovine mastitis in Vietnam. The virulence genes encoding adhesion, siderophore, Shiga-toxin-producing, and antimicrobials resistant were first reported in Vietnam with low prevalence and contributed to the pathogenesis.

## Introduction

Mastitis is the inflammation of the mammary tissues during infection. It has been reported in numerous mammalian species, including domestic dairy cattle [1]. This disease affects herds in all countries and is an economically burdensome disease encountered by dairy farmers [2]. The average failure cost of bovine mastitis is estimated at $147 per cow per year, contributing to milk production losses and culling [3]. Decreasing milk production accounts for approximately 70% of the total cost of mastitis [4], and an estimated 60–70% of all antimicrobials administered to dairy farms are for preventing and treating mastitis [5].

*Escherichia coli* is the most frequently found Gram-negative pathogen during mastitis. It invades the udder through the teat, proliferates, and initiates an inflammatory response in dairy cows. It can be found in the environment surrounding dairy cows, such as the herd bedding, especially in wet conditions [6]. This disease affects high-yielding cows and may cause several yearly deaths in the most severe inflammation cases [7]. *Escherichia coli* is a facultatively anaerobe, rod-shaped, flagellated, and Gram-negative bacteria belonging to the Enterobacteriaceae family. This bacterium is found in the healthy gastrointestinal tract of humans and ruminants, including milk-producing animals, whereas most strains of E. coli are harmless commensals; however, some can cause diseases [8]. *Escherichia coli* mastitis in dairy cattle is diagnosed in a wide range of severity, from mild (local inflammatory changes in the mammary glands) to severe (significant systemic derangement) [9]. Many virulence factors of *E. coli* causing udder infection were investigated, including extended-spectrum β-lactamase (ESBL)-producing [10], antibiotic resistance and virulence genes [11], and genes coding Shiga-Toxin-Producing [12–14].

Although *E. coli* was the predominant bacteria isolated from the samples, there is a lack of knowledge concerning this pathogen and its virulence factors. In Vietnam, to the best of our knowledge, there is no study demonstrating the virulence factors in *E. coli* causing dairy mastitis. In a previous study, we reported that the prevalence of bovine mastitis in Vietnam was 2.9% monthly, and the isolation of *E. coli* was 7.6% (unpublished data).

This study aimed to determine the antibiotic-resistant profile and identify the molecular characterization of some antimicrobials and virulence genes of *E. coli*. The results of the present study would be helpful for either farmers or veterinarians to consider and/or make decisions to minimize the loss during and after episodes of mastitis caused by *E. coli*.

## Materials and Methods

### Ethical approval

Examinations and sample collection were performed according to the standard diagnosis measures without unnecessary animal harm. Approval from the Institutional Animal Ethics Committee was not required; the study did not affect the animals in excess of therapy.

### Study period and location

The study was conducted from March to September 2022 on six farms in the Nghe An province of Vietnam.

### Herds’ profile and sample

The animal population consisted of Holstein-Friesian dairy cows from intensively managed, fully housed, and total mixed ration-fed. The average herd size was 3,000 milking cows. All milking cows were kept in free-stall barns with manure recycled solid as bedding and milked 2–3 times per day. Five calves were kept in nursery sheds and weaned; heifers were kept in loose-barn style sheds. Six hundred and eighty-six samples were collected from clinical mastitis cases (diagnosed by veterinarians at the farms with abnormalities in udders, such as swelling, heat, hardness, redness, pain, and the milk appearance, i.e., watery, flakes, clots, or pus) under aseptic conditions (sterilized sample vials, clean udder(s), sampling, labeling, refrigeration post-collection, and transport to the lab in the icebox), and cultured on the day of arrival.

### Isolation of the target pathogen

To isolate *E. coli*, a loopful (10 μL) of samples was first cultured on blood agar (incubated at 37°C for 24–48 h); the samples were culture-positive if one or more colonies were observed (≥100 Colony-forming unit/mL) [15], Gram-negative, rod-shaped checked by Gram-stain and microscope, and inoculated on MacConkey agar (Oxoid, UK) and Chromatic agar (Liofilchem, Italy) (incubated at 37°C for 24 h). A dry, pink-to-red colony on MacConkey agar and a pink-reddish-mauve colony on Chromatic agar were selected for biochemical tests for confirmation. The biochemical tests for *E. coli* include indole production (Oxoid), citrate utilization (Oxoid), motility (Oxoid) [16], and other tests, including carbohydrate fermentation (glucose, sucrose, and lactose) and gas production (Oxoid), methyl-red and Voges-Proskauer (Oxoid), oxidase (BD, USA), and catalase (Samchun, Korea). All isolates came from clinical mastitis cases from dairy farms in north-central Vietnam. Because we were only interested in antimicrobial resistance and its related genes together with virulence genes, we did not investigate the prevalence of this pathogen and its source (e.g., bedding materials, milking machine, and water).

### Antimicrobial susceptibility test

The antibiogram studies of the conformed *E. coli* species were conducted by disk diffusion test (DDT) with the Mueller-Hinton agar (MHA) (Oxoid) technique, described in Clinical and Laboratory Standards Institute (CLSI) M02-A11 [17]. First, the bacteria were spread over the surface of sterile MHA plates using a sterile cotton swab, and antibiotic disks (Oxoid) (Table-1) were placed over the surface of inoculated plates. The plates were dried on a vertical surface for 15 min and incubated at 37°C for 16–24 h. The zone of inhibitions of each antibiotic was recorded in millimeters (mm) and corresponded to the CLSI standard values of respective antibiotics. Multidrug resistance (MDR) was an acquired non-susceptibility to at least one agent in three or more antimicrobial categories [18]. The antimicrobial agents used in the study are described in Table-1.

**Table-1:** The antimicrobials used for this study.

Antimicrobial	Abbreviation	Code	Concentration
Ceftiofur	EFT	EFT30	30 μg
Gentamicin	GEN	CN120	120 μg
Sulfamethoxazole/Trimethoprim	STX	SXT25	1.25/23.75 μg
Imipenem	IMI	IPM10	10 μg
Nalidixic acid	NAL	NA30	30 μg
Trimethoprim	TRI	W5	5 μg
Piperacillin	PIP	PRL100	100 μg
Enrofloxacin	ENR	ENR5	5 μg
Florfenicol	FLO	FFC30	30 μg
Ciprofloxacin	CIP	CIP5	5 μg
Tetracycline	TET	TE30	30 μg
Colistin sulfate	COL	CT10	10 μg
Polymyxin B	POL	PB300	300 μg
Streptomycin	STR	S10	10 μg
Amoxycillin	AMO	AML10	10 μg
Sulfonamides	SUL	S3 300	300 μg
Ampicillin	AMP	AMP10	10 μg
Sulfamethoxazole	SUX	RL25	25 μg
Lincomycin	LIN	MY15	15 μg

EFT=Ceftiofur, GEN=Gentamicin, STX=Sulfamethoxazole/Trimethoprim, IMI=Imipenem, NAL=Nalidixic acid, TRI=Trimethoprim, PIP=Piperacillin, ENR=Enrofloxacin, FLO=Florfenicol, CIP=Ciprofloxacin, TET=Tetracycline, COL=Colistin sulfate, POL=Polymyxin B, STR=Streptomycin, AMO=Amoxycillin, SUL=Sulfonamides, AMP=Ampicillin, SUX=Sulfamethoxazole, LIN=Lincomycin

### Extended-spectrum β-lactamase production

Extended-spectrum β-lactamase production tests followed CLSI M100-30^th^ [19] using the disk-diffusion method with MHA. Two sets of antibiotics (Liofilchem) were used: Ceftazidime (30 μg) with Ceftazidime-clavulanate (30/10 μg) and Cefotaxime (30 μg) with Cefotaxime-clavulanate (30/10 μg). The inoculum was prepared according to the standard disk-diffusion procedures. The agar plate was incubated at 35°C ± 2°C for 16–20 h. The results were interpreted as ESBLs positive when the inhibition zone of either antimicrobial agent tested in combination with clavulanate was bigger than the zone diameter of the agent when tested alone over 5 mm. The criteria for interpreting the test results are demonstrated in Table-2.

**Table-2:** Conditions to confirm ESBL-producing *Escherichia coli*.

Single antibiotic diameter zone	Combination diameter zone
Ceftazidime <22 mm	Ceftazidime-clavulanate ≥27 mm
Cefotaxime <27 mm	Cefotaxime-clavulanate ≥32 mm

ESBL=Extended-spectrum β-lactamase

### Polymerase chain reaction (PCR)

DNA extraction using the Chelex 100 Resin (Sigma, Germany) protocol [20]: Colonies on the agar were collected and enriched in peptone water overnight; the medium (1.5 mL) was centrifuged in 800 × *g* for 5 min at 4°C. The supernatant was collected (500 μL) and centrifuged at 13,000× *g* for 5 min at 4°C; the supernatant was removed, followed by the addition of 200 μL of Chelex 100 (in TE buffer [TBR, Vietnam]), and mixed well. Subsequently, the mixture was incubated at 56°C with shaking at 800 rpm for 30 min, vortexed for 10 seconds, and incubated at 96°C for 8 min at 800 rpm. The vial was vortexed and centrifuged at 13,000× *g* for 3 min at 4°C. Around 100 μL of supernatant was used for PCR analysis. DNA extracted (sample) was stored in an Eppendorf tube and stored at −20°C until the PCR reaction was carried out.

#### Molecular confirmation of the isolates

The preliminary confirmed *E. coli* isolates were subjected to confirmation by genotyping using *E. coli*-specific gene *mal*B [21]. Briefly, a PCR reaction mixture was adjusted to 20 μL (3 μL nuclease-free PCR water (Hach, US), 10 μL mastermix (MyTaq HS Red Mix, [Bioline, US]), 1 μL each forward and revert primers (Table-3) [21–32], and 5 μL DNA templates). DNA extracted from *E. coli* strain ATCC25922 (Microbiologics, US) and *Salmonella enteritidis* strain ATCC13076 (Microbiologics) was used as the positive and negative controls, respectively. A 100–1500 bp DNA ladder (PCRBIO Ladder IV [PCRbio, UK]) was used. Amplification was conducted with an initial denaturation at 95°C for 3 min followed by 35 cycles of 95°C for 15 s, 58°C for 15 s, and 72°C for 15 s on a thermal cycler (Biometra TProfessional, Germany). The PCR products were purified and identified by agar electrophoresis (Mupid-Exu, US) and transillumination (Major Science - MBE 200A, Taiwan).

**Table-3:** Oligonucleotide primers used for amplification of the various targeted genes in *Escherichia coli*.

Gene	Primer sequences (5’- 3’)	Annealing temp.	Size (bp)	Reference
*Confirmation*				
*mal*B				
F	CTTTATCGGCCCTCACTCAA	58	585	[21]
R	AGGTGCTCATCATGGGAAAG			
*β-lactamases*				
*bla*_SHV_				
F	CTTTATCGGCCCTCACTCAA	53	237	[22]
R	AGGTGCTCATCATGGGAAAG			
*bla*_KPC_				
F	CGTCTAGTTCTGCTGTCTTG	56	798	[23]
R	CTTGTCATCCTTGTTAGGCG			
*bla*_OXA48_				
F	TTGGTGGCATCGATTATCGG	54	744	[24]
R	GAGCACTTCTTTTGTGATGGC			
*Sulfonamides*				
*sul*1				
F	TTCGGCATTCTGAATCTCAC	50	822	[25]
R	ATGATCTAACCCTCGGTCTC			
*sul*2				
F	CGGCATCGTCAACATAACC	54	722	[26]
R	GTGTGCGGATGAAGTCAG			
*Tetracycline*				
*tet*A				
F	GGTTCACTCGAACGACGTCA	57	577	[27]
R	CTGTCCGACAAGTTGCATGA			
*tet*B				
F	CCTCAGCTTCTCAACGCGTG	56	634	[25]
R	GCACCTTGCTGATGACTCTT			
*Trimethoprim*				
*DHFR-I*				
F	AAGAATGGAGTTATCGGGAATG	56	391	[26]
R	GGGTAAAAACTGGCCTAAAATTG			
*Quinolone*				
*qnr*A				
F	ATTTCTCACGCCAGGATTTG	54	516	[28]
R	GATCGGCAAAGGTTAGGTCA			
*Multidrug efflux pump*				
*acr*A_Kp_				
F	ATTTCTCACGCCAGGATTTG	55	940	[29]
R	GATCGGCAAAGGTTAGGTCA			
*Adhesion*				
F41				
F	GCATCAGCGGCAGTATCT	53	380	[30]
R	GTCCCTAGCTCAGTATTATCACCT			
F5				
F	TATTATCTTAGGTGGTATGG	47	314	[30]
R	GGTATCCTTTAGCAGCAGTATTTC			
Intimin				
F	ATATCCGTTTTAATGGCTATCT	52	425	[30]
R	AATCTTCTGCGTACTGTGTTCA			
*Shiga-toxin producing*				
*stx*				
F	GAACGAAATAATTTATATGTG	47	907	[31]
R	GATTTGATTGTTACAGTCAT			
*st×2*				
F	GTGCCTGTTACTGGGTTTTTCTTC	52	118	[30]
R	AGGGGTCGATATCTCTGTCC			
*Iron acquisition system*				
*iut*A				
F	GGCTGGACATCATGGGAACTGG	61	302	[32]
R	CGTCGGGAACGGGTAGAATCG			
*iro*N				
F	AAGTCAAAGCAGGGGTTGCCCG	63	665	[32]
R	GACGCCGACATTAAGACGCAG			

#### Confirmation of drug-resistance genes in the isolates

*Escherichia coli* isolates were confirmed with some specific genes corresponding to antibiotic resistance, including *tet*A and *tetB* associated with tetracycline resistance, *sul*1 and *sul*2 for sulfonamide (SUL) resistance, *bla*SHV and *bla*KPC for β-lactamases production, *bla*OXA48 encoding carbapenemase, *qnr*A for quinolones, and DHFR-I for trimethoprim (TRI) resistant. The reaction mixture was prepared, and the tests followed the protocol described above with primers, sequences, predicted sizes, and annealing temperatures described in Table-3.

#### Detection of virulence factors

The virulence factors associated with *E. coli* were determined by targeting specific genes, *stx* and *stx2*, for encoding Shiga-toxin, and adhesion genes responsible for colonization, including F5, F41, and Intimin (*eae)*, and siderophore-encoded genes (*iro*N and *iut*A). The reaction mixture was prepared, and the tests followed the protocol described above with primers, sequences, predicted sizes, and annealing temperatures described in Table-3.

### Statistical analysis

This study used spreadsheets as software tools for data entry, storage, analysis, and visualization.

## Results and Discussion

### The distribution and confirmation of *E. coli* in the samples

**Figure-1:**
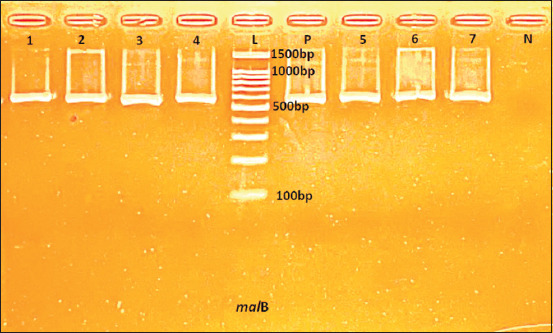
Polymerase chain reaction assay result for *Escherichia coli* identification by *mal*B (585bp) gene; DNA ladder (L); positive control (P), negative control (N), samples (1–7).

During the study period, six hundred and eighty-six samples were sent to the Laboratory (belonging to TH Milk Food joint stock company with ISO/IEC 17025:2017 certification number VILAS 1047). The isolation and identification process followed the protocols of the Laboratory. Fifty isolates were preliminarily identified as *E. coli* by biochemical reactions and confirmed as *E. coli* by PCR of the *mal*B gene (Figure-1).

## Antimicrobial susceptibility results

**Figure-2:**
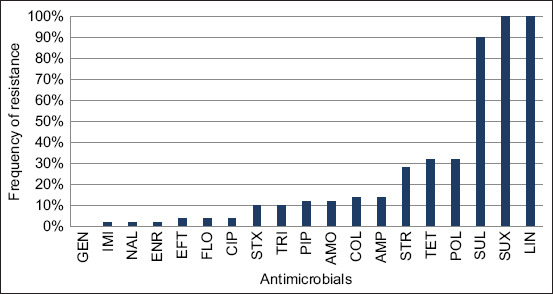
Drugs resistance proportion of *Escherichia coli* isolates against antibiotics used. GEN=Gentamicin, IMI=Imipenem, NAL=Nalidixic acid, ENR=Enrofloxacin, EFT=Ceftiofur, FLO=Florfenicol, CIP=Ciprofloxacin, STX=Sulfamethoxazole/Trimethoprim, TRI=Trimethoprim, PIP=Piperacillin, AMO=Amoxycillin, COL=Colistin sulfate, AMP=Ampicillin, STR=Streptomycin, TET=Tetracycline, POL=Polymyxin B, SUL=Sulfonamides, SUX=Sulfamethoxazole, LIN=Lincomycin.

All *mal*B gene positives were processed for antibiogram studies. Nineteen common antibiotics belonged to nine classes, and resistance levels varied between antibiotics (Figure-2). All isolates were fully resistant to lincomycin (LIN) and sulfamethoxazole, followed by SUL with 90% resistance. The resistance to tetracycline (TET) and polymyxin (POL) was 32%, whereas Streptomycin was 28%. Resistance against ampicillin (AMP) and colistin sulfate was 14%, while resistance for amoxicillin (AMO) and piperacillin (PIP) was 14% for each drug, followed by TRI and sulfamethoxazole/trimethoprim (STX) with 10% resistance. The lowest percentage of the resistance group was 4% against ciprofloxacin (CIP), florfenicol, and ceftiofur (EFT). It was revealed that out of fifty isolates, only one (2%) was resistant to enrofloxacin, nalidixic acid, and imipenem (IMI), and none were resistant to gentamicin (GEN). Our study found the highest resistance of *E. coli* belonging to LIN, STX, and SUL. Even though LIN has not been used in the farms before, especially to treat animals with mastitis, thus we did not investigate the resistance gene related to this antimicrobial. The resistance toward LIN and STX in our study was similar to findings in China (LIN [98.8%], STX [53%]) [33]. The resistance rates toward TET were 57.4% in Iran [25], 45.4% in Jordan [34], 10%–34.8% in China [11, 35], and 14.6% in Switzerland [36], which is quite different from our results. Antimicrobial resistance to ampicillin can be used to predict the susceptibility to amoxicillin [37]; thus, the resistance to AMP and AMO in our study was 14% and 12%, respectively, which was lower than a finding of 100% and 79.5% in China [38], these antimicrobials often used in the farms to treat not only mastitis but also other diseases, which could be the reason for the resistance occur. Our results showed that resistance to PIP, GEN, and CIP was lower than a study in China, which reported 36.9%, 18.5%, and 8.7% resistance, respectively [11]. In contrast, similar results of GEN and CIP were reported in Canada [39] and northern China [40]. Imipenem has not been used in the farms but showed high sensitivity in our study; this finding is similar to a study in China which claimed that none of the *E. coli* isolates was resistant to IMI [38]. Ceftiofur-EFT, a third-generation cephalosporin, is one of the most used antibiotics in the dairy industry, with a dose of 1 mg/kg body weight generally results in a 0-hour withdrawal time for the milk in dairy cows [41], which is why this antimicrobial was widely considered to use in dairy farms. In our study, we found that resistance toward this drug is relatively higher than in studies in Switzerland (1.2%) [36], France (1.4%) [42], and Canada (2.6%) [43].

### Multidrug resistance in *E. coli*

Of 19 antibiotics used, one isolate showed no sensitivity to 12 different drugs, and none were resistant to only one specific drug. Most were resistant to three (14/50) or four (13/50) drugs. The 50 isolates resistant to 2–12 different drugs are presented in 19 patterns (Table-4). Twenty-seven strains were resistant to at least two antibiotic classes (54%), and multidrug resistance (resistance to ≥3 antimicrobial classes) was 46% (twenty-three strains) of the total isolates tested (Table-4). Increasing drug-resistance in animal pathogenic bacteria is problematic for veterinary treatment and is risky for humans. It was previously reported that the MDR proportion was 12.7%, 37.1%, 79.5%, 84.2%, and 38.4% isolates in Canada, Jordan, Iran, Bangladesh, and China, respectively, from clinical mastitis samples [11, 12, 34, 43, 44]. This alarming finding indicated relatively high levels and the possible association between antimicrobial resistance widespread among bacterial strains from animals and from humans to animals and the overuse of antibiotics to deal with animal health issues. Therefore, farm antibiotic management is essential for controlling and preventing MDR risk.

**Table-4:** Antimicrobial resistance patterns of *Escherichia coli* isolates.

Antimicrobials resistant pattern	Number of drugs	Number of antimicrobial classes	Number of isolates
AMP-SUX-FLO-TET-SUL-STX-AMO-LIN-POL-TRI-SUX-STR	12	6	1
AMP-SUX-TET-SUL-STX-AMO-LIN-COL-TRI-SUX-STR	11	5	1
AMP-SUX-TET-SUL-STX-AMO-LIN-TRI-SUX-STR	10	5	3
AMP-SUX-FLO-SUL-AMO-LIN-POL-SUX-STR	9	5	1
AMP-SUX-ENR-SUL-CIP-LIN-NAL-POL	8	4	1
SUX-TET-SUL-LIN-COL-POL-STR	7	4	2
SUX-TET-SUL-LIN-POL-STR	6	4	3
SUX-SUL-EFT-LIN-POL	5	3	1
SUX-TET-SUL-CIP-LIN	5	4	1
SUX-TET-SUL-LIN-STR	5	4	1
SUX-TET-SUL-LIN-COL	5	3	1
SUX-SUL-LIN-POL-STR	5	3	2
SUX-SUL-LIN-POL	4	2	5
SUX-SUL-LIN-COL	4	2	3
SUX-SUL-EFT-LIN	4	3	1
SUX-TET-SUL-LIN	4	3	3
SUX-SUL-IMI-LIN	4	3	1
SUX-SUL-LIN	3	2	14
SUX-LIN	2	2	5
Total			50

AMP=Ampicillin, SUX=Sulfamethoxazole, ENR=Enrofloxacin, FLO=Florfenicol, TET=Tetracycline, SUL=Sulfonamide, STX=Sulfamethoxazole/Trimethoprim, EFT=Ceftiofur, CIP=Ciprofloxacin, AMO=Amoxycillin, IMI=Imipenem, LIN=Lincomycin, COL=Colistin sulfate, NAL=Nalidixic acid, POL=Polymyxin, GEN=Gentamicin, TRI=Trimethoprim, PIP=Piperacillin, STR=Streptomycin

### Extended-spectrum β-lactamase production in *E. coli*

Extended-spectrum β-lactamase production was performed for all isolates, and none of these *E. coli* strains were positive for ESBL. To the best of our knowledge, this study reported the first and most extensive screening of ESBL-producing *E. coli* causing cattle mastitis published in Vietnam. None of the isolates were ESBL producers, which was supported by a similar study in Canada (0%) [45] and in France with a prevalence of 0.3% (5/1745) [46]. On the other hand, other studies in Greek [10], China [47], and Egypt [48] reported that 6.7%, 23.5%, and 100% *E. coli* isolated from dairy farms were ESBL producers, respectively. Therefore, farms and veterinarians should focus on good hygiene practices and the prudent use of antimicrobial agents against diseases affecting dairy cows, especially mastitis treatments, to maintain similar levels of ESBL-producing bacteria.

### Antibiotic resistance gene detection

**Figure-3:**
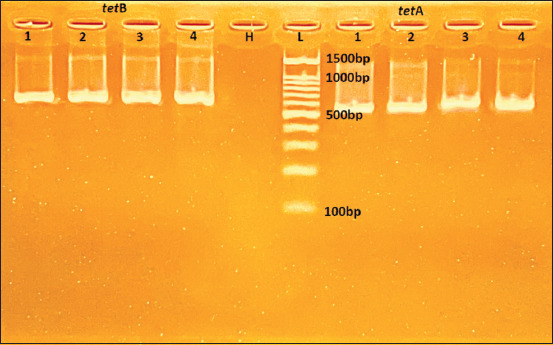
Polymerase chain reaction result for *tet*B (634bp) and *tet*A (577bp) gene, DNA ladder (L), H_2_O for negative control (H).

The antimicrobial resistance profile is helpful for understanding the pathogenesis of *E. coli* infection in bovine mastitis [49]. Some studies have shown that the *tet*A gene is more prevalent than the *tet*B gene in *E. coli* strains [11, 44, 50, 51], and our results (Table-5) were different with similar proportions in both *tet*A and *tet*B (Figure-3). Our study revealed that six isolates that carried *tet*A were tetracycline-resistant, similar to the isolates that carried *tet*B, but none of the tetracycline-resistant isolates harbored both these genes. This result supported the agreement between genotype and phenotype. Sulfonamides were the first drugs acting selectively on bacteria that could be used systemically. Today, they are infrequently used, in part due to widespread resistance. The target of SUL, and the basis for their selectivity, is the enzyme dihydropteroate synthase in the folic acid pathway [52]. Our study found that 26% and 30% of isolates carried *sul*1 and *sul*2 genes (Table-5), which was supported by a study in Bangladesh with 47.1% and 32.4% abundance, respectively [51]. The resistance to β-lactam antibiotics in pathogens from livestock has been continuously reported in recent years [53, 54], and predominantly ESBLs produced from *E. coli* are considered a key resistance mechanism [55]. Recent studies have claimed that the resistant genes with Beta-lactam antibiotics, included TEM [a beta-lactamase named from an Athenian patient (Temoniera)], KPC *(Klebsiella pneumonia*e carbapenemase), and OXA (Oxacillinase) with prevalence from 6.4% to 98.7% [11, 25, 33, 40, 44, 47, 54]. Interestingly, our study has not found any isolate that harbored these genes (Table-4), which is different and need future studies to discover the underlying reasons. Similar to Beta-lactam resistance-related genes, the current study has not found any isolate carrying DHFR-I and *qnr*A genes related to TRI and quinolone resistance (Table-5), which is different from a study in China with 95.6% resistance to quinolones [11]. The results revealed the differences between genotype and phenotype in SUL-resistant *E. coli* strains. One of the factors is the misuse of antimicrobials, but probably not the main or single factor involved in antimicrobial resistance [56].

**Table-5:** Antimicrobial resistance genes identified in *Escherichia coli* isolates from mastitis cows.

Virulence genes	Total isolates tested	Positive isolates	Positive proportion (%)
*bla*SHV	50	0	0
*bla*OXA48	50	0	0
*bla*KPC	50	0	0
*tet*A	50	6	12
*tet*B	50	6	12
*DHFR-I*	50	0	0
*sul*1	50	13	26
*sul*2	50	15	30
*qnr*A	50	0	0

### Virulence genes of *E. coli* isolates

**Figure-4:**
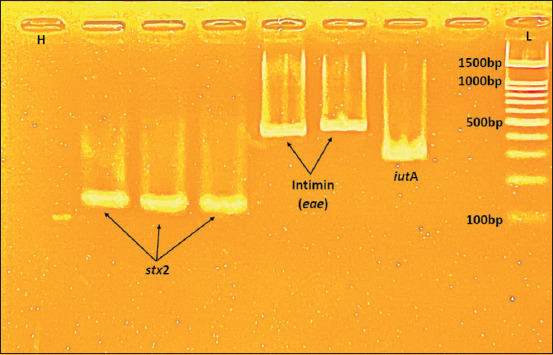
Polymerase chain reaction result for *iut*A (302bp) gene, positive sample (1), *eae* gene (425bp), *stx2* gene (118bp) negative sample (2–4), DNA ladder (L), negative control (H-H_2_O).

The virulence profile coded by different genes is an essential factor correlated with the pathogenesis of *E. coli* infections in bovine mastitis. The virulence factors, such as adhesion (F5, F41, and Intimin), provide colonization ability to the pathogens in mammary cells as the first step of infection; these genes were the most prevalent virulence factors identified in clinical bovine mastitis cases [57]. Shiga-toxin producing *E. coli* (STEC; verotoxin-producing *E. coli* (VTEC)) have been associated with bovine intramammary infections (IMI) [14], encoded by the *stx1* and *stx2* genes [58]. The iron uptake systems in *E. coli*, including different siderophores (encoded by *iro*N, *iut*A, and other genes), have been widely studied and are known as the primary mechanism of ferric acquisition [59]. From fifty isolated strains, we performed a PCR analysis to detect adhesion (F5, F41, and Intimin), siderophores (*iro*N and *iut*A), and Shiga-toxin genes (*stx, stx1*, and *stx2*). Only two (4%) isolates harbored Intimin, one was positive with *iut*A, and 3/50 isolates (6%) carried *stx2* genes from these strains (Figure-4). The rest of the genes were not detected (Table-6).

**Table-6:** Prevalence of virulence genes identified in *Escherichia coli* isolates.

Virulence genes	Total isolates tested	Positive isolates	Positive proportion (%)
*F41*	50	0	0
*F5* (*K99*)	50	0	0
*Intimin* (*eae*)	50	2	4
*iutA*	50	1	2
*iroN*	50	0	0
*st×1*	50	0	0
*st×2*	50	3	6

In similar studies, it was reported that 100% of adhesion factors were found in Brazil [60], 64% of Intimin genes from *E. coli* were found in Jordan, 10.3% in Iran [12], and 81% in China [11]. Our results varied from these findings; it could indicate that *E. coli* causing mastitis were not Enterotoxigenic *E. coli* strains. Unfortunately, siderophores were not extensively studied; some reports claimed that virulence factors *iut*A (10.6%) and *iro*N (14.3%) were found in Iran [61], 20.7% of *iut*A factor was isolated from mastitis samples found in Switzerland [36]. Our results are different from these findings; they revealed that this virulence factor might not play an essential role in the pathogenesis of *E. coli* and needs future investigations for other siderophore-related genes. Shiga-toxin production was widely reported. Studies in Iran indicated that genes encoding Shiga-toxins (*stx1* and *stx2*) were the most prevalent virulence factors that were isolated from the clinical bovine mastitis cases 77.8% and 13.9%, respectively [25]; 64.1% and 12.8% isolates had *stx2* and both *stx1-stx2*, respectively [12], 31% isolates contained *stx1* in New Zealand [62]. Compared to these findings, our results differed significantly from the reports obtained from Bangladesh and China, where these genes were not found in *E. coli* isolated from mastitis cases [11, 35].

## Conclusion

This study demonstrated that *E. coli* strains isolated from bovine mastitis cases in Vietnam do not carry major adhesion, siderophore, or Shiga-toxin-producing encoding genes. Most isolates are multidrug-resistant, resulting from the overuse of antimicrobials to control mastitis or other diseases at the farms. The high prevalence of multidrug-resistant *E. coli* is alarming and indicates a potential risk for mastitis treatment and human health. Our study demonstrated that all *E. coli* strains were not ESBL producers, confirmed by the DDT results and lack of encoding genes. Tetracyclines and SUL should be reduced in clinical treatment because the resistance level and the prevalence of related genes were confirmed. The results from this study provided information that supports dairy farmers/owners and veterinarians to improve the usage and management of antimicrobials during mastitis treatment, especially with *E. coli* mastitis in large-scale herds.

## Authors’ Contributions

TTM and DXB: Designed the study, analysis and interpretation of the data, and drafted the manuscript. LVT, VDM, and BTPM: Collected samples and performed experiments. VMD and DTML: Conducted the PCR and drafted and revised the manuscript. All authors have read, reviewed, and approved the final manuscript.
